# Eurasian lynx fitness shows little variation across Scandinavian human-dominated landscapes

**DOI:** 10.1038/s41598-019-45569-2

**Published:** 2019-06-20

**Authors:** José Vicente López-Bao, Malin Aronsson, John D. C. Linnell, John Odden, Jens Persson, Henrik Andrén

**Affiliations:** 1 Research Unit of Biodiversity (UO/CSIC/PA), Oviedo University, Mieres, 33600 Spain; 2 Grimsö Wildlife Research Station, Department of Ecology, Swedish University of Agricultural Sciences, SE-73091 Riddarhyttan, Sweden; 3 Department of Zoology, Stockholm University, SE-10691 Stockholm, Sweden; 4 Norwegian Institute for Natural Research, PO Box 5685 Torgard, NO-7485 Trondheim, Norway

**Keywords:** Conservation biology, Population dynamics

## Abstract

Despite extensive research on the ecology and behavioural adaptations of large carnivores in human-dominated landscapes, information about the fitness consequences of sharing landscapes is still limited. We assessed the variation in three consecutive components of female fitness: the probability of reproduction, litter size and juvenile survival in relation to environmental and human factors in a solitary carnivore, the Eurasian lynx (*Lynx lynx*), occurring in human-dominated landscapes in Scandinavia. We used demographic data from 57 radio-collared adult females between 1995–2011 (126 radio-years). Overall, the yearly probability of female reproduction was 0.80, mean litter size was 2.34 (range 1–4) and the probability to find a female that reproduced in the spring being accompanied by at least one offspring during the subsequent winter was 0.70. We did not find evidence that food availability was a key factor influencing female fitness. Female lynx may adapt to food availability when establishing their home ranges by adopting an obstinate strategy, ensuring a minimum amount of prey necessary for survival and reproduction even during periods of prey scarcity. In human-dominated landscapes, where sufficient prey are available for lynx, mortality risk may have a larger influence on lynx population dynamics compared to food availability. Our results suggest that lynx population dynamics in human-dominated landscapes may be mainly driven by human impacts on survival.

## Introduction

In a human-dominated world^1^, an increasing number of large carnivore populations occur in human-dominated environments^2–4^, and there is accumulating evidence showing the ability of these species to persist in such landscapes^5,6^. Factors affecting large carnivore distribution, population dynamics, and their flexibility in habitat selection or diet in human-dominated landscapes have attracted growing attention^7–15^. Moreover, multiple consequences of sharing landscapes, such as human-induced stress, prevalence of diseases, or impacts on genetic structure, have also been highlighted^16–18^. However, despite extensive research focused on large carnivores persisting in human-dominated landscapes, information about the fitness consequences of sharing landscapes, or the existence of ecological traps – a mismatch between habitat quality and fitness – is still limited^19^.

Key resources for animal species are seldom distributed evenly across landscapes, leading to demographic variation in space^20,21^. At the landscape scale, the heterogeneous distribution of human activities creates mosaics of factors influencing large carnivore persistence^22–24^, such as heterogeneous distributions of food availability or mortality risk factors^25–29^. Such spatial heterogeneity may have important consequences for population dynamics, triggering spatial patterns in carnivore occupancy, fitness and, ultimately, carnivore persistence. For example, some large carnivores can supplement their diets with anthropogenic food sources^26,30^, which may increase their fitness or may buffer oscillations in natural prey availability^26^. Prey species, moreover, can take advantage of human activities. Wild ungulates can benefit from agriculture and forestry, which may result in higher densities of prey available for large carnivores in modified habitats^13,29,31–34^. Prey species can also reduce predation risk by moving closer to areas with higher human presence, which some carnivores avoid (i.e., “human shield effect”)^35–37^. On the other hand, humans are the primary cause of large carnivore mortality, driving population dynamics and carnivore recovery^38–41^, and a growing attention has emerged on how humans impact carnivore survival in relation to habitat^28,29^.

A trade-off for large carnivores may emerge between the potential demographic cost (mortality) and benefits (food availability) of sharing landscapes. Thus, it is expected that animals will balance their choices to reduce fitness consequences, balancing between access to food resources and mortality risks^19,29^. In order to get a comprehensive perspective of the impact of humans on large carnivore persistence in human-dominated landscapes, it is crucial to understand how space use, and consequently habitat selection, will affect fitness by relating environmental variation and human-related factors with different fitness components.

**Figure 1:**
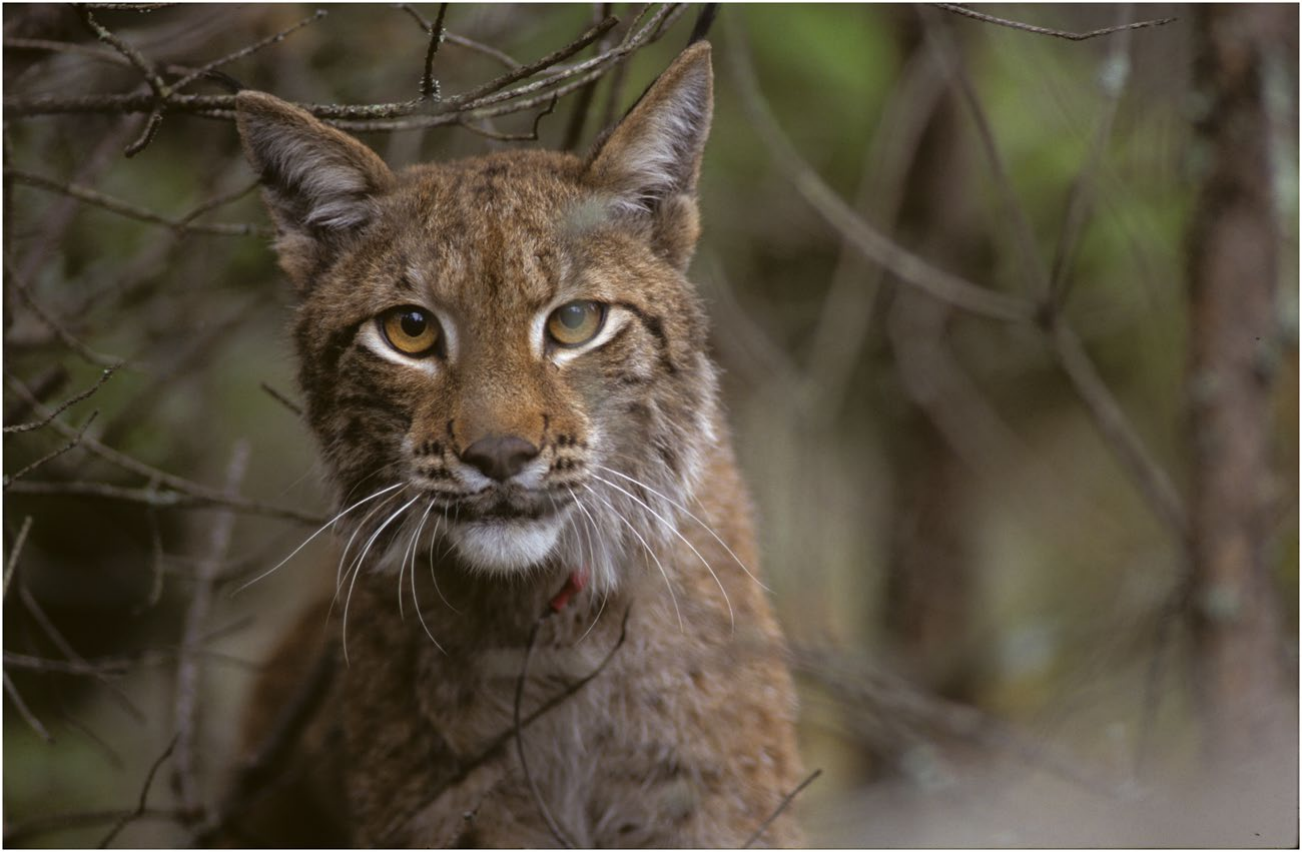
Eurasian lynx *(Lynx lynx)*. Picture courtesy of Henrik Andrén.

In this study, we assessed variation in consecutive fitness components in a solitary large carnivore, the Eurasian lynx (*Lynx lynx*) (Fig. 1), hereafter lynx, occurring in multi-used landscapes in Scandinavia. Lynx shows tolerance to human activities^2,11,42^, provided that there is good vegetation cover (i.e., forested areas)^43,44^. Lynx are a seasonal breeder^45^ that give birth in late May/early June^46^ to between 1 and 4 kittens (optimal litter size of 2)^47^, and about half of juveniles survive their first year^40,48,49^. Starvation is considered a potential factor influencing kitten/juvenile lynx survival^40^. Roe deer (*Capreolus capreolus*) represent the main prey of lynx in southern Scandinavia^50,51^, although domestic sheep *(Ovis aries)* can comprise an important fraction of the diet in some areas in Norway during the summer^52^. Roe deer abundance is correlated with agricultural lands^31,33,53^. Humans are the main cause of lynx mortality (i.e., hunting, poaching, vehicle collisions)^40^. Still, lynx frequently use human-dominated areas^33^, although they actively avoid humans at a fine scale. For example, reproductive females select den sites in remote and rugged terrain^54^ and avoid risky habitats with higher densities of roe deer during the early kitten rearing phase^13,33^.

We focused on three consecutive components of lynx female fitness in relation to environmental variation and human-related factors within their home ranges: (1) probability of reproduction, (2) litter size, and (3) juvenile survival to the first winter. Firstly, we explored how different proxies of food availability within female home ranges influenced the probability of lynx reproduction. We predicted that if reproduction constitutes an important investment for lynx females, the probability of reproduction should be positively influenced by food availability^24^. Secondly, we assessed whether litter size was influenced by food availability. Although it could be expected that energetic costs for females would be the driving factor determining litter size^55^, in Scandinavia, lynx litter size does not vary according to female body mass, reproductive category or among years, and a litter size of 2 kittens is associated with higher fitness than both smaller and larger litters^47^. Therefore, we predicted that in the case of Eurasian lynx, food availability should not influence litter size. Finally, we evaluated the influence of environmental and human factors on juvenile lynx survival. We expected that factors associated with food availability should positively influence survival, whereas the opposite pattern should be observed for human-related variables^29,40^.

## Methods

### Study areas

This research was performed in two study areas of the south-central Scandinavian Peninsula, located in Sweden and Norway (57°–63° N, 9°–17° E). The Norwegian study area consists of several parallel river valleys running from north to south separated by hills, and with a north-south altitudinal gradient, from 200 to 800 m.a.s.l. in the north to <300 m.a.s.l. in the south. The Swedish part, on the other hand, is less hilly with altitude ranging from 50 to 500 m.a.s.l. The forests in both zones are managed for pulp and timber, which creates a forest mosaic of even-aged forest stands. In both countries, there is a north-south environmental gradient where primary productivity, proportion of agricultural land, human density, roe deer harvest and road density increase towards the south, whereas time with snow cover and snow depth increase towards the north.

### Lynx capture and monitoring

We used data from 57 radio-collared potentially breeding female lynx (≥2 years)^48^, monitored for a total of 126 radio-years between 1995–2011 within the Scandinavian Lynx Project, Scandlynx. The minimum age at capture was known for all lynx previously tagged with microchip at natal den sites. We classified previously unmarked animals as either subadult (<18 months) or adult (>18 months) based on whether they were structurally fully grown. Furthermore, young animals captured in spring, while still travelling with their mothers, were classified as <1 year old. Age was also determined retrospectively post-mortem by counting incremental lines in the tooth cementum^56^. In our dataset, lynx females were between 2 and 13 years old and were monitored, on average, for 2.2 years (range: 1–8 years).

Lynx were live-captured using walk-through box-traps, foot-snares placed at fresh kills, or treed with the use of dogs. Captured lynx were immobilised with a mixture of ketamine (5 mg/kg) and medetomidine (0.2 mg/kg)^57^. Animals were fitted with VHF-transmitters (from 1996–2010: VHF-collars Mod.335 and Mod.400NH, Telonics, USA), intra-peritoneal transmitters (IMP/150/L and IMP/400/L, Telonics, USA) or GPS-collars (2003–2012: GPS plus mini, Vectronic Aerospace, Germany; Lotek 3300SL, Lotek Wireless, Canada; Televilt Posrec 300 and Tellus 1 C, Followit, Sweden).

VHF-collared lynx were located from the ground or from the air at least two to four times per month. GPS-collared animals were relocated at least once every day. Reproductive females were localized more frequently during the birth period because female lynx movement patterns can indicate whether they had given birth^49,58^. Once a potential breeding female was detected, the natal den site was visited between 2 and 6 weeks after estimation of birth to verify reproduction, and record litter size. Natal dens were checked significantly (Mann-Whitney U test, *P* = 0.004; n = 89) later in Norway (26.3 ± 11.7 SD days) than in Sweden (19.3 ± 11.4 SD days). Lynx females adopt a central place foraging behaviour centred on the den site for the first 6–8 weeks of the kittens’ lives. Therefore, it was almost impossible to have failed to detect a reproductive event where kittens survived the perinatal period.

Without extensive radio-tracking or camera trapping efforts it is difficult to get information on kitten survival during the first snow-free season. However, after snowfall, we used observations of snow tracks from radio-collared female lynx to assess the number of kittens that accompanied her, and thus had survived to the onset of winter (between November and January). As the hunting season starts for lynx on February 1 (Norway) or March 1 (Sweden), this means that hunting did not affect juvenile survival (although the influence of poaching during that time cannot be excluded)^40^. Furthermore, juvenile lynx tend not to disperse before February to March^59^, so there is little risk that dispersal would be misinterpreted as mortality in our study.

### Space use, environmental and human factors

Female lynx home ranges (km^2^) were estimated using the fixed-kernel method with the *‘adehabitatHR’* package^60^ in R^61^. Lynx home ranges were estimated as the 90% kernel-isopleth using the reference bandwidth multiplied by 0.8^62^, starting from February 1 to January 31 the next year. The number of locations acquired per individual varied extensively as radio-tracking technology developed during the study period (VHF *vs*. GPS). To reduce biases in home range size estimations due to different sampling frequencies among animals and years, we randomly sampled 1 location/day per individual^63^. Only animals in years with ≥25 locations collected during ≥7 months were used^62^. In our case, this resulted in a total of 126 annual home ranges, where the mean (±SD) number of annual locations and monitoring months per female were 80.5 ± 63.2 and 10.2 ± 2.6, respectively. For each home range, latitude and longitude coordinates were calculated at the centroid, in order to explore for spatial patterns in the three selected fitness components.

We used *roe deer harvest* (number of roe deer shot/10 km^2^ per year) as a coarse proxy for roe deer density. Roe deer harvest data is available at hunting district level from the Swedish Hunters Association for hunting and wildlife management (www.jagareforbundet.se and www.viltdata.se), and at the municipality level in Norway, from Statistics Norway (www.ssb.no). Roe deer hunting bag statistics can be considered a good functional proxy for roe deer density on lynx home range scales, as it is related to several other measurements of roe deer density^62,64–66^. For each lynx annual home range, we calculated the roe deer harvest the same year and the year before as the area-weighted average annual roe deer bag size across the hunting districts or municipalities overlapping each home range. Roe deer harvest ranged between 0.49 to 108 shot roe deer per 10 km^2^. Data on *sheep density* (number of heads/km^2^) was obtained from the Swedish Board of Agriculture (www.jordbruksverket.se) and Statistics Norway (www.ssb.no), at the municipality level. Sheep are available for lynx mainly from April to October within our study areas. We calculated sheep density within each annual home range as the area-weighted average density across overlapping municipalities. Sheep density ranged between 0.11 and 6.81 heads/km^2^.

We calculated the *proportion of agricultural lands*, *coniferous forests* (all types pooled) and *deciduous forests* (all types pooled) within each lynx home range. This aggregation was made considering the functional structure of each vegetation type in relation to refuge and food availability for large carnivores^43,44,67–69^. Land cover was obtained from a 25 × 25 m digital land cover map for Sweden, and a 20 × 20 m digital land cover map for Norway (Swedish Land Cover [SMD], National Land Survey of Sweden; Northern Research Institute’s vegetation map, Norway). Within lynx home ranges, the proportion of agricultural lands ranged from 0 to 0.45, whereas the proportion of coniferous and deciduous forest ranged from 0.30 to 0.80, and from 0.02 to 0.37, respectively. We also calculated the mean *elevation* and *roughness* for each home range based on a 25 × 25 m Digital Elevation Model (Geographical Data Sweden, Lantmäteriet; Norge digital, Statens kartverk, Norway). Mean *elevation* (m) was calculated by averaging elevations of all 25 × 25 m raster cells included in each lynx home range, whereas topographic *roughness* (m) was considered as the standard deviation of the elevations of all 25 × 25 m raster cells, in order to quantify topographic heterogeneity. The elevation of lynx home ranges ranged between 53 and 788 m.a.s.l. and roughness ranged between 13 and 270 m.

We also measured several indicators of human activities and disturbance within lynx home ranges. Data on *human density* was obtained from the Swedish National Institute of Statistics (www.scb.se) and Statistics Norway (www.ssb.no) at the municipality level, and calculated as the area-weighted average human density across municipalities overlapping each home range. At the lynx home range level, mean (±SD) human population density was 37.0 ± 46.6 inhabitants per km^2^ (range 1.4–234.7). Secondly, by using the 25 × 25 m digital land cover map we calculated the proportion of *urban areas* within lynx home ranges (i.e., settlements, urban and industrial areas pooled). This proportion was very small at the home range level ranging from 0 to 0.07 only. We also measured the length of primary and secondary roads. Primary roads included public roads (European, national, county and municipal roads), which are most often paved. Secondary roads, on the other hand, included all unpaved and forest roads. Road data was obtained from the Swedish Transport Administration database (www.trafikverket.se) and N50 kartdata, Statens kartverk, Norway (www.kartverket.no). We calculated the *density of primary and secondary roads* by dividing the length of each road type by the total area of the lynx home ranges. Mean (±SD) primary and secondary road densities were 0.98 ± 0.76 and 0.97 ± 0.39 km/km^2^, respectively (ranges: 0.09–2.43 and 0.15–2.22).

All the female lynx studied here persisted in a human-dominated landscape outside high-level protected areas, such as national parks. So, we did not consider the level of landscape protection in our analyses.

### Lynx family groups

Because local variations in lynx abundance influence lynx spatial behaviour^62^, it may also affect the outcome of the different fitness components through competition. We therefore included a covariate in our analyses – *lynx family groups* - to control for potential effects of competition on fitness components. We were focused on females because of intra-sex competition^70,71^. We used the national lynx monitoring results from Sweden and Norway where lynx family groups (i.e., females with kittens) are estimated at a regional scale based on snow tracking in January and February each year^72^. We calculated *lynx family groups* as the area-weighted annual average number of lynx family groups across the biogeographical regions (Sweden) or carnivore management areas (Norway) overlapping each annual lynx home range. Lynx family group densities varied between 0.15 and 3.91 family groups per 1,000 km^2^.

### Statistical analyses

We built three different sets of Generalized Linear Mixed Models (GLMMs) using the *“lme4”* package^73^ to test for variation in lynx female fitness components in relation to environmental and human factors (i.e., probability of reproduction, litter size, and juvenile survival). Each set of candidate models considered different predictors according to the specific hypothesis tested. For each fitness component, we started by considering the full model including all predictors of interest. All predictors were standardized. Afterwards, we built a subset of competing models using a backward procedure from the full model. Furthermore, all models included three random factors: study area, individual identity, and year. The magnitude of multicollinearity among predictors was assessed using the variance inflation factor (VIF), excluding those predictors with VIF > 5 (i.e., deciduous forest and elevation were excluded, and primary and secondary roads were pooled to satisfy this criteria). We used the “*car*” package to calculate the Wald *χ*^2^ to evaluate the significance levels for selected model parameters^74^. AICc weights (*w*_*i*_) were estimated using the “*MuMIn*” package^75^. Within each set of candidate models, univariate models from the best candidate model were also calculated. We checked for overdispersion in all Poisson error distribution models, and it was unnecessary to account for it in any case.

Firstly, we built a model-set for lynx female reproduction. This dataset contained 117 cases (that is, 117 radio-years). We used GLMMs with binomial error distribution and logit-link function, and a binomial response variable (i.e., 1 for reproduction and 0 for no reproduction). The following predictors were considered in this model-set: *proportion of agricultural land*, *proportion of coniferous forest*, *roughness*, *sheep density, lynx family groups* and *roe deer harvest*. We only included *roe deer harvest* the same year in our analyses because roe deer harvest the same year and the year before were highly correlated (Pearson correlation, r_P_ = 0.92, *P* < 0.0001). Moreover, *roe deer harvest* the same year best explained the probability of lynx female reproduction (AICc^76^ was 123.3 for roe deer harvest the same year *vs*. 124.0 for roe deer harvest the year before. Individual identity, year and study area were included as random factors in both models).

Secondly, we built a model-set evaluating variation in litter size, and for this fitness component our dataset contained 83 cases (i.e., non-reproducing females were not considered). We used GLMMs with a Poisson error distribution and log-link function, and litter size (count) as the response variable. The same predictors as for the previous fitness component (probability of reproduction) were used.

Thirdly, we built a model-set evaluating juvenile survival through the first winter following birth. For this fitness component, our dataset contained 79 cases (for 4 cases in the litter size dataset there was no reliable information on survival in the first winter). We used GLMMs with a Poisson error distribution and log-link function, and litter size in winter as a response variable. The number of kittens detected in summer were included as an offset in all models. For this model-set we added predictors associated with two factors affecting lynx survival: food availability and mortality risk. For food availability, we used the following predictors: *roe deer harvest*, *proportion of agricultural land*, *proportion of coniferous forest*, *sheep density* (this predictor can represent both food availability and mortality risk due to conflicts associated to lynx depredation on livestock) and *roughness*. For mortality risk, we used *urban areas*, *human population density*, and *road density (primary roads* and *secondary roads* were pooled). We did not consider *Lynx family groups* in this model-set due to convergence issues.

Finally, we additionally built two separate sets of GLMM models in order to explore for spatial patterns in the fitness components evaluated in this study. Firstly, following the same model structure explained above (all models included three random factors: study area, individual identity, and year), we tested for the existence of spatial patterns in fitness components using *latitude* and *longitude* as predictors. Afterwards, and with the aim to facilitate the interpretation of our results, we also evaluated differences in the three selected fitness components between study areas (i.e., countries; in these GLMM models study area was treated as a fixed factor). All data and scripts used here are available to other researchers upon request.

### Ethic statement

Lynx are a protected species in Sweden under the EU Habitats Directive (Annex II and IV), and animals were captured and immobilized using strict handling protocols^77^. This research and handling protocols for lynx were approved by the Swedish and Norwegian Animal Ethics Committees (permits C275/95, C16/0, Dnr 410–5531–98 Nf).

## Results

Based on our dataset, the annual probability of lynx female reproduction was 0.80 (n = 117), and this probability was not influenced by female age (GLMM with the probability of lynx reproduction as response variable, *age:* single and quadratic terms, *roe deer harvest*, and individual identity, year and study area as random factors; *age*: *χ*^2^ = 0.03, d.f. = 1, *P* = 0.865; *roe deer harvest*: *χ*^2^ = 2.05, d.f. = 1, *P* = 0.152; n = 69). On the other hand, although the yearly probability of reproduction for ≥ 3 years old females (all pooled) was 0.81 (n = 47) (0.90 considering 3 years old females only; n = 11), compared to 0.77 for 2 years old females (n = 22), these differences were not significantly different either (GLMM with the probability of lynx reproduction together with *roe deer harvest* as covariates, and individual identity, year and study area as random factors; effect of *Age group* (group 1: 2 years old females and group 2: ≥ 3 years old females): *χ*^2^ = 0.66, d.f. = 1, *P* = 0.416; n = 69).

The most parsimonious model explaining the probability of female lynx reproduction included *roughness* and *sheep density* (*w*_*i*_* = *0.48; Table 1). The probability of female lynx reproduction was significantly and negatively influenced by both factors (Table 2). Furthermore, an additional model including the *proportion of agricultural land* within home ranges, together with the two predictors mentioned above, was within an ΔAICc < 2 (ΔAICc = 1.4), although its support decreased by half (*w*_*i*_ = 0.25; Table 1). The *proportion of agricultural land* showed a positive, but non-significant, influence on the probability of reproduction (Table 2). *Agricultural land* within lynx home ranges was positively correlated with *roe deer harvest* (Pearson correlation, r_P_ = 0.54, *P* < 0.001), and *roughness* was negatively and significantly correlated with *agricultural land* and *roe deer harvest* (Pearson correlations, r_P_ = −0.49 and −0.75, respectively, *P* < 0.001). On the other hand, there was no correlation between *sheep density* and *roe deer harvest* in our dataset (Pearson correlation, r_P_ = 0.01, *P* = 0.908). In addition, although the average *roughness* value ( ± SD) in our dataset was 79.8 ± 72.4, *roughness* was significantly higher (GLM, *χ*^2^ = 74.67, d.f. = 1, P < 0.001) in Norway (a mean value of 143) than in Sweden (a mean value of 64). Similarly, *sheep density* did not vary between Norway and Sweden (GLM, *χ*^2^ = 0.48, d.f. = 1, P = 0.486).

**Table 1:** Competing Generalized Linear Mixed Models explaining Eurasian lynx probability of reproduction in central-south Scandinavia in relation to variation in environmental and human factors within female lynx home ranges.

COMPETING MODELS:	AICc	ΔAICc	*w*_*i*_
Roughness + Sheep density	119.3		0.48
Roughness + Sheep density + Agricultural land	120.7	1.4	0.25
Roughness	121.6	2.3	0.15
Roughness + Sheep density + Agricultural land + Coniferous forest	123.0	3.6	0.08
Roughness + Sheep density + Agricultural land + Coniferous forest + Roe deer harvest	125.3	6.0	0.02
Null model	127.1	7.8	0.01
Roughness + Sheep density + Agricultural land + Coniferous forest + Roe deer harvest + Lynx family groups	127.7	8.4	0.01
**Nested models from the best candidate model:**			
Roughness	121.6		0.53
Sheep density	121.9	0.3	0.47

Models are ranked based on AICc, difference in AICc relative to the highest-ranked model (ΔAICc) and AIC-weights (*w*_*i*_).

**Table 2:** Parameter estimates (±SE) for the selected models with ΔAICc < 2 explaining Eurasian lynx reproduction in central-south Scandinavia in relation to variation in environmental and human factors within female lynx home ranges.

Parametric coefficients	Estimate (±SE)	*P*
Best candidate model:		
***Roughness*** + ***Sheep density***		
*Intercept*	1.49 ± 0.25	
Roughness	−0.49 ± 0.22	0.028
Sheep density	−0.46 ± 0.23	0.047
Alternative model:		
***Roughness*** + ***Sheep density*** + ***Agricultural land***		
*Intercept*	1.52 ± 0.26	
Roughness	−0.30 ± 0.29	0.308
Sheep density	−0.59 ± 0.27	0.028
Agricultural land	0.35 ± 0.40	0.375

Mean litter size was 2.32 (median = 2, range 1–4; n = 83). In accordance with our predictions, the null model (intercept-only model) was the best model explaining variability in litter size in the Eurasian lynx (*w*_*i*_* = *0.46; Table 3), implying that none of our variables helped explain variation in litter size. We observed a second model with an ΔAICc < 2, the model including *roe deer harvest*, although its support was lower (*w*_*i*_ = 0.35; positive but non-significant effect: estimate (±SE) = 0.09 ± 0.07, *P*-value = 0.185; Table 3).

**Table 3:** Competing Generalized Linear Mixed Models explaining Eurasian lynx productivity (litter size) in central-south Scandinavia in relation to variation in environmental and human factors within female lynx home ranges.

COMPETING MODELS:	AICc	ΔAICc	*w*_*i*_
Null model	253.0		0.46
Roe deer harvest	253.5	0.5	0.35
Roe deer harvest + Lynx family groups	255.5	2.5	0.13
Roe deer harvest + Lynx family groups + Sheep density	257.8	4.8	0.04
Roe deer harvest + Lynx family groups + Sheep density + Coniferous forest	260.3	7.3	0.01
Roe deer harvest + Lynx family groups + Sheep density + Coniferous forest + Agricultural land	262.8	9.8	0.004
Roe deer harvest + Lynx family groups + Sheep density + Coniferous forest + Agricultural land + Roughness	265.4	12.4	0.001

Models are ranked based on AICc, difference in AICc relative to the highest-ranked model (ΔAICc) and AIC-weights (*w*_*i*_).

The probability to observing a female lynx that reproduced in the spring accompanied by at least one offspring the following winter was 0.70 (n = 79). In 54% of these cases (30 out of 55), all offspring survived. On average, for litter sizes of 1 (n = 5), all the offspring survived the following winter, whereas this figure decreased to 81% and 72% for litter sizes of 2 and 3, respectively. However, we did not detect a significant influence of litter size on the proportion of offspring surviving until the following winter (Kruskal-Wallis test considering litter sizes from 1 to 3, d.f. = 2, *P* = 0.084; n = 54. There was only one case of a litter size of 4 in our dataset). The most parsimonious model explaining the probability of juvenile survival to the following winter was the null model (*w*_*i*_ = 0.34; Table 4). Three additional models were within ΔAICc < 2 (Table 4), which included different combinations of *roughness*, *proportion of agricultural land*, and *roe deer harvest* (Tables 4 and S1). All predictors in these models were non-significant excepting *roughness*, which showed a positive impact on juvenile survival (Table S1).

**Table 4:** Competing Generalized Linear Mixed Models explaining juvenile lynx survival in central-south Scandinavia in relation to variation in environmental and human factors within home ranges.

COMPETING MODELS:	AICc	ΔAICc	*w*_*i*_
Null model	219.2		0.34
Roughness	219.6	0.4	0.27
Roughness + Agricultural land	220.4	1.2	0.18
Roughness + Agricultural land + Roe deer harvest	221.1	1.9	0.13
Roughness + Agricultural land + Roe deer harvest + Sheep density	222.9	3.7	0.05
Roughness + Agricultural land + Roe deer harvest + Sheep density + Road density	224.9	5.7	0.012
Roughness + Agricultural land + Roe deer harvest + Sheep density + Road density + Urban areas	227.4	8.2	0.005
Roughness + Agricultural land + Roe deer harvest + Sheep density + Road density + Urban areas + Human density	230.0	10.8	0.001

Models are ranked based on AICc, difference in AICc relative to the highest-ranked model (ΔAICc) and AIC-weights (*w*_*i*_).

We did not find significant spatial patterns in the variation of the three fitness parameters studied (Table S2), although we observed that the model containing *longitud*e and *latitude* showed a lower AICc value in the case of juvenile survival (a ΔAICc > 2: 217.1 *vs*. 219.2; Tables 4 and S1). Similarly, there were no significant differences in fitness parameters between countries, with the exception of juvenile survival being higher in Norway (GLMMs with individual identity and year as random factors and the effect of study area on: probability of lynx reproduction, *P* = 0.154; litter size, *P* = 0.199; juvenile survival, *P* < 0.001). The probability to observing a female lynx that reproduced in the spring accompanied by at least one offspring the following winter was 0.61 in Sweden (n = 46) and 0.81 in Norway (n = 33). *Longitude* was negatively and significantly correlated with *roughness* (Pearson correlation, r_P_ = −0.80, *P* < 0.001).

## Discussion

Most female lynx studied in this human-dominated landscape reproduced every year. Reproduction was nearly constant regardless of the level of food availability within lynx home ranges. Since we confirmed lynx reproduction several weeks after parturition (mean number of days = 21.8 ± 11.9 SD), kitten mortality in the first three weeks of life could influence the observed yearly probability of female reproduction (0.80). But, even so, the bias would have been underestimating this probability. Roe deer represent the main prey species for lynx in our study areas^50,51^, although lynx also feed on sheep in some places^52^. However, we did not observe an impact of *roe deer harvest* on the probability of lynx reproduction. This result suggests that lynx reproduction is relatively insensitive to prey availability across the range of deer densities observed here. This is probably due to the efficient hunting behaviour of lynx (i.e., lynx show a Type II functional response in kill rate of roe deer^50,78^), and/or, alternatively, due to a low level of energetic investment placed by lynx into gestation (but see^79^).

*Roughness*, however, showed a negative and significant effect on the probability of lynx reproduction. At fine scales, when resting or killing prey, for example, lynx select rugged areas with a medium degree of human modification (e.g., a mix of forest and agriculture lands) to avoid high levels of human activity^11^. On the other hand, roe deer abundance is correlated with agricultural lands^31,33,53^, with the highest densities observed in fragmented landscapes where they benefit from open forests, crops, and Supplementary feeding sites^11,13,32^. In fact, in our dataset *agricultural land* within lynx home ranges was positively correlated with *roe deer harvest* and the probability of female reproduction was positively influenced by the *proportion of agricultural land* within home ranges – although not significant (Table 2). At the home range level, the probability of lynx reproduction was lower towards higher *roughness* values (Tables 2 and S3) and, although we did not observe a significant spatial variation in this fitness parameter, this result was probably partly influenced by the highest rugged landscape from one of the study areas, Norway (Table S3). For high *roughness* values all cases of no reproduction were in Norway (Table S3). *Roughness* within home ranges was negatively and significantly correlated with *agricultural land* and *roe deer harvest*, which may influence reproduction in some particular rugged landscapes or contexts.

Similarly, *sheep density* showed a negative and significant effect on the probability of lynx reproduction. There is no correlation between *sheep density* and *roe deer harvest* in our dataset and *sheep density* did not vary between Norway and Sweden, although their availability for lynx may differ between countries (in Sweden sheep are fenced, whereas they are mainly free-ranging in Norway). Therefore, the observed negative effect of sheep density seems to not be related with low roe deer availability for lynx.

Highest kill rates of sheep by lynx have been observed in areas with low roe deer density^52^. Although the management of lynx differs between Sweden and Norway (i.e., lynx population goals and hunting quotas), hunting / culling is carried out in both countries. If culling is focused on areas with higher levels of conflict (i.e., lynx attacks on sheep), high turn-over of adult females (replaced by younger individuals) could potentially explain our findings. Compared to adult females (≥3 years old), two-year old females have shown a lower breeding proportion in different study sites across Scandinavia^46^, including this study (the yearly probability of reproduction for ≥3 years old females was 0.81, n = 47, whereas this figure was 0.77 for 2 years old females, n = 22). In Norway, the predicted mean age of harvested lynx females was 3.1^80^. Therefore, the removal of 3 years old females may have a slight influence on breeding proportions. Whether other factors (predation before we checked natal dens^81^ or the availability of other sources of food for lynx) may influence the probability of lynx reproduction deserves further investigation.

As we predicted, litter size was not affected by any of the environmental factors explored. The lack of influence of the different surrogates of food availability used here supports the idea that the level of prenatal investment in female lynx is low^46,47^. The energetic cost for female lynx to produce between 1 to 4 kittens is similar, and females do not allocate important amounts of energy during gestation^47^. A litter size of around 2 has been suggested as the optimum litter size for the Eurasian lynx in different environmental conditions and across variation in individual attributes^46^.

Similar to other large carnivore species, human-caused mortality is the most important source of mortality for lynx^29,40^. When we evaluated the influence of human-related factors and food availability on juvenile survival, we found again that food availability within the female home ranges did not influence juvenile survival. Actually, none of the variables used here helped explain variation in juvenile survival properly, as the null model was selected as the best candidate model. However, we observed a spatial pattern (between countries) in juvenile lynx survival, being higher in Norway than Sweden. The probability to observing a female lynx that reproduced in the spring accompanied by at least one offspring the following winter was 0.61 in Sweden (n = 46) and 0.81 in Norway (n = 33). The positive effect observed for *roughness* is in line with the idea that landscape attributes can influence lynx survival^29^. Previously, it has been shown how lynx select for rugged areas in order to avoid high levels of human activity^11,13,82^. However, different field sampling protocols may also explain the differences in juvenile survival between countries. If early kitten mortality is important, this could have influenced our results.

Overall, from the different environmental and human factors analysed, and fitness components evaluated, our results show how food availability was not a key factor influencing female fitness, and that different fitness parameters may be influenced by different factors. Although roe deer abundance increased in human-dominated landscapes, we did not find support that food availability was a key factor influencing the probability of lynx reproduction, litter size or juvenile survival. This result, together with female lynx behaviour becoming a central place forager during the breeding period^49^, suggests that when female lynx establish their home ranges, they cope with unpredictable changes in food availability by adopting an obstinate strategy, ensuring the presence of a minimum amount of prey which is necessary for survival and reproduction^62,71^ (lynx are efficient hunters, even at low prey densities^50,78^). This is supported by the fact that non-reproducing females do not decrease their home range size during summer (when food availability is higher), whereas reproducing females do due to their restricted movement around den sites^62^.

In human-dominated landscapes, where sufficient prey are available for lynx, mortality risk may have a larger influence on lynx dynamics compared to food availability. Different species may have their demography differentially influenced by pressures on different life cycle stages. Contrary to ungulates, where variation in reproduction and juvenile survival drive dynamics^83^, in the Eurasian lynx, our results suggest that variation in reproduction, litter size and juvenile survival may not be determinants for lynx dynamics. In contrast, survival after 10 months of age may well be a key driver (see Nilsen *et al*. 2009^84^ for an example on the importance of adult survival in roe deer populations exposed to harvest and predation). Therefore, lynx dynamics in human-dominated landscapes may be mainly driven by human impacts on survival.

## Supplementary information

Supplementary Information
