# Supplementary material for: Eurasian lynx fitness shows little variation across Scandinavian human-dominated landscapes
Source: Sci Rep. 2019 Jun 20;9:8903. doi: 10.1038/s41598-019-45569-2 (PMC6586631; doi:10.1038/s41598-019-45569-2)
Supplement: Supplementary file 1 — Supplementary Information [file 41598_2019_45569_MOESM1_ESM.docx]

Supplementary Information

**Eurasian lynx fitness shows little variation across Scandinavian human-dominated landscapes**

José Vicente López-Bao, Malin Aronsson, John D.C. Linnell, John Odden, Jens Persson and Henrik Andrén

**Table S1.** Parameter estimates (±SE) for the selected models, apart from the null model, with ΔAICc<2 explaining Eurasian lynx juvenile survival in central-south Scandinavia in relation to variation in environmental and human factors within female lynx home ranges.

| Parametric coefficients | Estimate (±SE) | *P* |
| --- | --- | --- |
|  |  |  |
| *Roughness* |  |  |
| *Intercept* | -0.64 ±0.10 |  |
| Roughness | 0.24 ±0.09 | 0.005 |
|  |  |  |
| *Roughness* + *Agricultural land* |  |  |
| *Intercept* | -0.65 ±0.10 |  |
| Roughness | 0.31 ±0.10 | 0.002 |
| Agricultural land | 0.14 ±0.11 | 0.196 |
|  |  |  |
| *Roughness* + *Agricultural land* *+ Roe deer harvest* |  |  |
| *Intercept* | -0.65 ±0.10 |  |
| Roughness | 0.21 ±0.13 | 0.117 |
| Roe deer harvest | -0.21 ±0.16 | 0.197 |
| Agricultural land | 0.20 ±0.12 | 0.087 |

**Table S2.** Parameter estimates (±SE) for the models explaining variation in Eurasian lynx fitness components in central-south Scandinavia in relation to the spatial location of home ranges.

| MODEL | Estimate (±SE) | *P* |
| --- | --- | --- |
| **Probability of female lynx reproduction** |  |  |
| *Intercept* | 1.39 ±0.24 |  |
| Longitude | 0.25 ±0.26 | 0.335 |
| Latitude | -0.19 ±0.28 | 0.476 |
| AICc = 127.9 |  |  |
|  |  |  |
| **Litters size** |  |  |
| *Intercept* | 0.83 ±0.07 |  |
| Longitude | 0.07 ±0.08 | 0.374 |
| Latitude | -0.02 ±0.08 | 0.773 |
| AICc = 255.0 |  |  |
|  |  |  |
| **Juvenile survival** |  |  |
| *Intercept* | -0.65 ±0.10 |  |
| Longitude | -0.22 ±0.12 | 0.067 |
| Latitude | 0.14 ±0.12 | 0.222 |
| AICc = 217.1 |  |  |

**Table S3.** Distribution of cases of “no reproduction” across the observed range of variation in *roughness* in our study areas. NO: Norway. SE: Sweden.

| *Roughness* class | N | Probability of reproduction | Cases no reproduction | |
| --- | --- | --- | --- | --- |
|  |  |  |  |  |
| 0-50 | 63 | 0.84 | 10 | NO: 0 / SE : 10 |
| 51-100 | 14 | 1.00 | 0 |  |
| 101-150 | 15 | 0.73 | 4 | NO: 4 / SE : 0 |
| 151-200 | 15 | 0.60 | 6 | NO: 6 / SE : 0 |
| >201 | 10 | 0.60 | 4 | NO: 4 / SE : 0 |
